# Biofabrication of Poly(glycerol sebacate) Scaffolds Functionalized with a Decellularized Bone Extracellular Matrix for Bone Tissue Engineering

**DOI:** 10.3390/bioengineering10010030

**Published:** 2022-12-25

**Authors:** Selcan Guler, Kian Eichholz, Farhad Chariyev-Prinz, Pierluca Pitacco, Halil Murat Aydin, Daniel J. Kelly, İbrahim Vargel

**Affiliations:** 1 Bioengineering Division, Institute of Science and Engineering, Hacettepe University, 06800 Ankara, Turkey; 2 Trinity Centre for Biomedical Engineering, Trinity Biomedical Sciences Institute, Trinity College Dublin, D02 R590 Dublin, Ireland; 3 Department of Mechanical and Manufacturing Engineering, School of Engineering, Trinity College Dublin, D02 R590 Dublin, Ireland; 4 Advanced Materials and Bioengineering Research Centre (AMBER), Royal College of Surgeons in Ireland and Trinity College Dublin, D02 F6N2 Dublin, Ireland; 5 Department of Anatomy, Royal College of Surgeons in Ireland, D02 YN77 Dublin, Ireland; 6 Department of Plastic and Reconstructive Surgery, Hacettepe University Hospitals, 06230 Ankara, Turkey

**Keywords:** poly(glycerol sebacate), bone tissue engineering, extracellular matrix, decellularization, osteogenesis, mesenchymal stem cells

## Abstract

The microarchitecture of bone tissue engineering (BTE) scaffolds has been shown to have a direct effect on the osteogenesis of mesenchymal stem cells (MSCs) and bone tissue regeneration. Poly(glycerol sebacate) (PGS) is a promising polymer that can be tailored to have specific mechanical properties, as well as be used to create microenvironments that are relevant in the context of BTE applications. In this study, we utilized PGS elastomer for the fabrication of a biocompatible and bioactive scaffold for BTE, with tissue-specific cues and a suitable microstructure for the osteogenic lineage commitment of MSCs. In order to achieve this, the PGS was functionalized with a decellularized bone (deB) extracellular matrix (ECM) (14% and 28% by weight) to enhance its osteoinductive potential. Two different pore sizes were fabricated (small: 100–150 μm and large: 250–355 μm) to determine a preferred pore size for in vitro osteogenesis. The decellularized bone ECM functionalization of the PGS not only improved initial cell attachment and osteogenesis but also enhanced the mechanical strength of the scaffold by up to 165 kPa. Furthermore, the constructs were also successfully tailored with an enhanced degradation rate/pH change and wettability. The highest bone-inserted small-pore scaffold had a 12% endpoint weight loss, and the pH was measured at around 7.14. The in vitro osteogenic differentiation of the MSCs in the PGS-deB blends revealed a better lineage commitment of the small-pore-sized and 28% (*w*/*w*) bone-inserted scaffolds, as evidenced by calcium quantification, ALP expression, and alizarin red staining. This study demonstrates a suitable pore size and amount of decellularized bone ECM for osteoinduction via precisely tailored PGS elastomer BTE scaffolds.

## 1. Introduction

Bone injuries and diseases arising from trauma, congenital anomalies, or tumor resections are treated with different surgical interventions. Bone graft materials, such as polymers, ceramic substitutes, and natural bone grafts (i.e., autografts, allografts, or xenografts), are widely used in the clinical treatment of bone defects [1,2,3]. Ceramics are known to be brittle [4,5], while natural graft tissues have issues in terms of limited material availability, additional surgical sites, rejection issues related to immunogenic incompatibilities [6], and potential risks of viral infections in xenotransplantation [7]. Bone tissue engineering (BTE) is an alternative strategy where other materials, such as polymers, can be utilized to aid bone tissue repair. The scaffolding approach is widely applied using different fabrication techniques, such as 3D printing, melt electro writing (MEW), solvent-casting particulate leaching, etc., based on the acquisition of the appropriate properties [8,9,10,11,12]. Since bone tissue formation is a multilateral process, several key criteria (mechanical, biological, and structural requirements) need to be considered when designing bone tissue engineering scaffolds [13]. Herein, utilizing polymeric scaffolds is advantageous as they can be tailored to mimic the native bone extracellular matrix (ECM) in terms of the appropriate mechanical, porosity, pore size, and inner-chemical properties [14]. The microarchitecture and chemical composition of these porous scaffolds have been shown to play a pivotal role in the regeneration of bone defects by providing cells with an appropriate microenvironment and tissue-specific cues to regulate the osteogenic lineage commitment of MSCs [15,16,17]. The importance of pore geometry, curvature, and, in particular, pore size, on the functionality of porous scaffolds is well documented. Specifically, pore size alone has been shown to affect stem cell fate in the context of bone tissue regeneration [15,18,19,20]. Although smaller pore sizes (100–135 μm) have been associated with enhanced cellular attachment due to the increased availability of binding sites, better vascularization was demonstrated in larger pore size (>300 μm) scaffolds [21]. In order to reveal the effect of pore size on bone tissue formation, many polymers have been investigated by themselves [22] or with their ceramic [23,24], polymer [25], or ECM composites [26]. There is no clear consensus on optimal pore size, as these values likely vary with fabrication method, cell type, and the material used.

Poly(glycerol-sebacate) (PGS) is a biodegradable elastomeric polymer obtained via a polycondensation reaction using equimolar glycerol and sebacic acid precursors followed by high-temperature crosslinking under a vacuum. Due to its tunable nature, which allows for the tissue-specific tailoring of its mechanical properties, degradation behavior, and hydrophilicity, this polymer has been employed for the replacement and regeneration of soft tissues, such as cardiovascular tissues [27,28], nerves [29], skin [30], cartilage [31] and tendons [32]. Recently, this material has been increasingly investigated for BTE applications [33,34,35]. The mechanical properties could be tuned for BTE application while maintaining elasticity [36]. From a surgical point of view, such elastic materials with sufficient mechanical strength would be beneficial; as such, scaffolds present an opportunity for surgeons to easily cut and reshape an off-the-shelf product for the patient during surgery. Besides its many advantages, one possible issue with such synthetic elastomers is that, even though PGS is biocompatible, like most synthetic polymers, it has limited bioactivity [37]. One way to improve the bioactivity of such a scaffold is with the incorporation of ECM components [38]. Decellularized bone matrix (DCB) is rich in organic components, such as collagen, proteoglycan, and bone growth factors (BMPs), as well as inorganic minerals (calcium phosphates) [39]. DCB is known to promote the osteogenic differentiation of MSCs and their attachment and proliferation [40]. In previous studies, various polymeric materials were functionalized with a DCB matrix, such as 3D-printed Poly-e-Caprolactone (PCL) [39,41,42,43,44] and electrospun bECM/PCL nanofibrous scaffolds [44].

The aim of this study was to functionalize a PGS elastomer, for the first time, with a decellularized bone matrix (DCB) and, thus, create an osteoinductive scaffold capable of supporting robust osteogenesis in bone marrow-derived MSCs. Additionally, this study also explored how the pore size within such scaffolds influences the osteogenic commitment of MSCs.

## 2. Materials and Methods

### 2.1. Generation of Decellularized Bone Powder

Decellularized bone powder was generated from bovine femoral condyles obtained from a local slaughterhouse. Briefly, cancellous bone fragments were minimized to smaller granules with the aid of a commercial coffee mill (Tefal, Ankara, Turkey). Grounded bone granules were stored at −80 °C until processing to preserve osteoinductive potential [45]. Defrosted bone granules were washed with phosphate-buffered saline (PBS) containing 0.1% (*w*/*v*) Ethylenediaminetetraacetic acid (EDTA) for 130 min at room temperature (RT) under shaking to roughly separate the excessive lipids from tissue. Separated lipids were discarded from the surface, and washing solution was replaced with fresh PBS containing 0.1% (*w*/*v*) EDTA and 10 mM Tris, kept at 4 °C overnight. Sodium dodecyl sulfate (SDS)-based decellularization procedure followed, and bone granules were decellularized with 0.5% SDS for 24 h at RT under shaking. Following decellularization, 3 days of PBS washing was applied to ensure the removal of the residual genomic material and excessive SDS from tissue. Decellularized bone granules were homogenized for 20 min to reduce the granule size and freeze-dried for 2 days to obtain the bone powder. The generated bone powder was sieved, and only a size between 45–50 µm was used.

### 2.2. Fabrication of PGS/deB Blend Scaffolds

**Figure 1:**
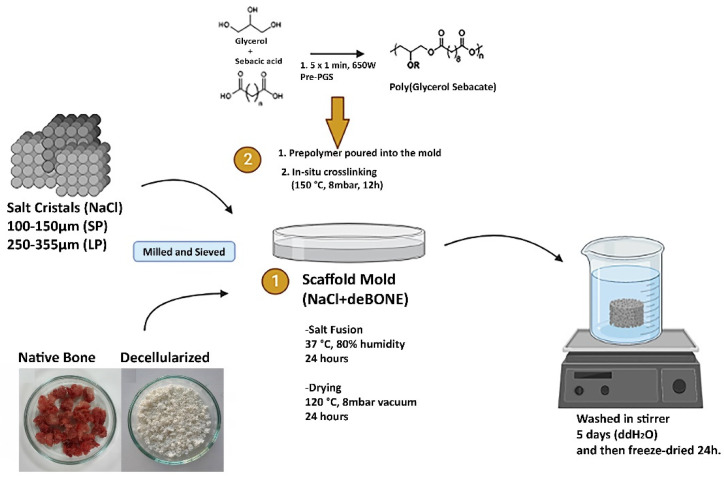
Schematic of the fabrication of the PGS/deB blend scaffolds. Stage 1 consisted of creating a scaffold template by blending the milled and sieved salt crystals (NaCl) (100–150 µm: small pores (SP) and 250–355 µm: large pores (LP)) with the decellularized bone powder followed by the in situ crosslinking of the prepolymer poured into the scaffold mold (Stage 2).

Scaffold molds were designed to determine the effect of both on the amount of bone incorporated in and the pore size on new bone tissue formation. For this purpose, scaffold templates were produced by blending milled and sieved salt crystals (100–150 µm: small pores (SP) and 250–355 µm: large pores (LP)) with the 14% and 28% (*w*/*w*) decellularized bone powder, which was allowed to fuse in an incubator at 37 °C and 90% humidity for 24 h followed by overnight low-vacuum drying at 120 °C. Microwave-irradiated pre-polymerization routes were followed [46], and accordingly, equimolar sebacic acid and glycerol were mixed in a glass Petri dish and were exposed to 650 Watt microwave 1 min × 5 repeats with 10 sec intervals. Viscous pre-PGS was dispensed in scaffold templates and was exposed to dehydrothermal crosslinking at 150 °C, 8 mbar vacuum for 12 h. Cured PGS/deB blends were immersed in ddH_2_O for 5 days by changing the washing solution every 24 h to obtain a porous scaffold by removing salt crystals from the structure. Scaffolds were finally freeze-dried at −80 °C overnight. Figure 1 shows the schematic representation of the fabrication of the PGS/deB blend scaffolds.

### 2.3. Characterization of PGS/deB Blend Scaffolds

*Physicochemical properties:* The chemical structure of the PS-deB blend scaffolds was characterized by attenuated total reflection Fourier transform infrared spectroscopy (ATR-FTIR, Agilent Technologies, USA). All samples were analyzed (ATR crystal: ZnSe) in the midinfrared region of 4000–650 cm^−1^ and recorded with a resolution of 1 cm^−1^. The organic content in the PGS/deB blend scaffolds and the onset of thermal degradation were analyzed with a thermogravimetric analysis (TGA) instrument (STA 1500, Perkin Elmer, Waltham, MA, USA). TGA analyses were performed in a nitrogen atmosphere with a temperature ranging between 10 to 1000 °C by changing 10 °C/min. For both the DSC/TGA analyses, the samples were weighed, ranging between 25 to 34 mg. The thermal behaviors of the PGS/deB blend scaffolds were evaluated with a differential scanning calorimeter (DSC) instrument (STA 1500, Perkin Elmer).

*Scaffold Microarchitecture:* The microarchitectures of all scaffolds were analyzed by scanning electron microscopy (SEM, Carl Zeiss Evo 50, Germany). Samples were coated with gold/palladium and imaged at an accelerating voltage of 20 or 30 kV. The cell-seeded scaffolds were also visualized with SEM Zeiss Ultra Plus. The cell-seeded constructs were fixed in 3% glutaraldehyde in 0.1 M cacodylate buffer at 4 °C overnight, and then the constructs were placed in 0.1 M cacodylate buffer (2 × 10 min). The scaffolds were dehydrated in a graded ethanol series: 50, 70, 90, and 100%, respectively, followed by immersion in hexamethyldisilazane (HMDS) (2 × 30 min). The fully dried scaffolds were mounted onto the SEM stubs and coated with gold/palladium for 30 s at 40 mA by using a Cressington 208 HR coater before the SEM observations at 5.00 kV were executed.

*Determination of density, porosity/water absorption, and pore size of scaffolds:* Prior to porosity determination, the scaffolds were fully dried via a freeze-drying method for 24 h at −80 °C. The dried porous PGS-deB blends were punched using an 8 mm cylindrical biopsy punch. For weight measurements, cylindrical specimens were weighed (W_0_) for each type of scaffold (*n* = 4) using a four decimal place balance (Sartorius, Göttingen, Germany), while the volume of each sample (*V*) was calculated following thickness (height; h) measurements by a digital caliper. The density of the scaffolds was calculated using the mass and volume of the scaffolds. To determine the porosity, the specimens were immersed in water at room temperature and their equilibrium mass was measured (We) when no further weight gain was observed, which was 17 h after immersion [30]. The porosity of all the types of scaffolds was calculated according to Equation (1):

(1) $$Porosity\;\left(\%\right)=\left(W_{e}-W_{0}\right)/\left(\rho .V\right)\times 100$$

where *Ρ* is the density of water at 25 °C (1 g/cm^3^). The water absorption of the scaffolds was evaluated by using We. For this purpose, the scaffolds were removed from the water after 17 h of immersion, and the excessive water around the scaffolds was carefully wiped off before the weight of the scaffolds was measured [47]. The water absorption ratio of the scaffolds was calculated according to Equation (2):

(2) $$Water\;absorption\;\left(\%\right):W_{loss}=\left(W_{e}-W_{0}\right)/\left(W_{0}\right)\times 100$$

where We is the equilibrium mass measured after 17 h of immersion in water and W_0_ is the initial dry weight of the scaffolds.

The average pore size of all PGS-deB scaffolds was measured by using Image J software. For this purpose, SEM micrographs were obtained from both the surface and cross-section of the scaffolds. The geometrically defined pores were selected for measurement (35 different pores were used for both the surface and cross-sectional regions).

*Degradation and pH change:* Porous scaffolds (Ø = 8 mm, h = 3 mm) were immersed in Tris-HCl buffer solution (pH = 7.4, 25 mL) and incubated at 37 °C under constant shaking for a period of time, while the pH values in the degradation solution were recorded for each of the defined time points. The percentage of weight loss was calculated by the following Equation (3):

(3) $$W_{loss}=(W_{0}-W_{t})/\left(W_{0}\right)\times 100$$

where W_0_ represents the initial dry weight before immersion, and Wt the dry weight measured at a defined time point. At each defined time point, the scaffolds were freeze-dried, and the complete dry weight of the scaffolds was measured. Degradation behavior was monitored for 28 days under dynamic conditions.

*Water contact angle:* All porous PGS-deB blend scaffolds’ water contact angles were measured with a contact angle analyzer (FTA125, First Ten Angstroms Inc., Portsmouth, VA, USA). Prior to testing, samples were prepared using a cylindrical 8 mm biopsy punch (*n* = 4). Contact angle degrees were determined with the sessile drop method in the air at room temperature via dosing with 5 µL water. Different wetting time points were selected (0, 30, 60, and 90 s), and the contact angles were measured.

*Mechanical Testing:* Cylindrical samples (diameter 8 mm) were mounted in a single-column Zwick/Roell universal testing machine (Hinsdale, IL, USA) with a 200 N load cell. Unconfined compression tests were performed on the scaffolds (*n* = 4), which were preloaded with 0.1 N and then compressed at a rate of 0.02% strain per second to a maximum of 10% strain. Compressive elastic modulus was calculated from the linear region of the stress-strain graphs, between the strain range of 4.8 and 6.4%.

*Cell Isolation and Expansion:* Bone marrow-derived mesenchymal stem cells were isolated from the femoral shaft of a freshly slaughtered 3-month-old pig obtained from a local abattoir, and the procedure was repeated as needed. MSCs were expanded in high-glucose Dulbecco’s modified Eagle medium (DMEM) GlutaMAX (Bioscience, Dublin, Ireland) supplemented with 10% fetal bovine serum (FBS, Biosciences, Dublin, Ireland) and 1% penicillin (100 U/mL)-streptomycin (100 μg/mL) (Biosciences, Dublin, Ireland) in a humidified atmosphere at 37 °C, 5% CO_2_. Expansion medium is abbreviated as XPAN. Tripotentiality was confirmed prior to use. pBMSCs were trypsinized and resuspended for a further passage at a density of 1 × 10^6^ cells/T175 flask, and the medium changed twice weekly.

*Osteogenic Differentiation Potential of isolated pBMSCs:* Osteogenic differentiation potential of isolated pBMSCs was determined for a 2D surface. For this purpose, pBMSCs (P4) were seeded at a density of 1 × 10^5^ cells per well (~1 × 10^3^ cells/cm^2^) in 6-well plates (*n* = 3). The upper 3 wells were labeled as positive and the bottom wells as negative. The cells were cultured with XPAN medium till cells reached 80% confluency. After that point, the positive wells were cultured with osteogenic induction medium, while the negative wells were kept cultured with the same XPAN medium. Osteogenic induction medium was obtained by adding 100 nm dexamethasone, 10 mM β-glycerolphosphate, and 50 μg/mL ascorbic acid into the XPAN medium. The culture medium was renewed every three days during osteogenic induction. Both positive and negative wells were stained with alizarin red solution (ARS, 1%, *w*/*v*) (Sigma-Aldrich, A5533, St. Louis, MI, USA); the pH of the solution was adjusted to 4.1 using 0.5 N ammonium hydroxide (Sigma-Aldrich). The pH of the ARS solution was checked each time before use. Monolayer cells were fixed with 100% iced ethanol for 10 min at RT, and then each well was washed with PBS. Cells were stained with ARS for 2 min and immediately afterwards were visualized using an inverted microscope, and macroscopic images were also taken.

*Cell-Seeding onto PGS/deB Blends and Osteogenic Induction:* Scaffolds were prepared individually via 5 mm diameter cylindrical punches and were subjected to ethylene oxide (EtO) sterilization. Before use, the scaffolds were kept at RT for 2 days to allow for complete dissipation of EtO. Single Agarose wells (3% (*w*/*v*) Agarose (Sigma-Aldrich)) were prepared for each scaffold to enhance seeding efficiency and also to prevent the detachment of the cells from the scaffolds by keeping them stabilized in the agarose well to avoid undesired floating in the medium [48,49]. Single molds were obtained with the aid of an 8 mm cylindrical punch. Prior to cell seeding, the scaffolds were placed in agarose wells and were kept in a 10% FBS-supplemented growth medium for 24 h in order to deposit protein-rich serum to enhance cell attachment to the scaffolds. Porcine bone marrow-derived stem cells (pBMSCs) were used between passages 2 and 4 (P2-P4) for cell culture studies. Cells were cultured in a standard expansion medium (DMEM Glutamax, 10% FBS, and 1% Pen-Strep). A density of 0.5 × 10^6^ pBMSC/scaffold was seeded onto individual scaffolds suspended in 30 μL of expansion media. Cells were allowed to adhere to the scaffolds by keeping them in normoxic conditions at 37 °C for 3 h. Following cell adhesion, a 2 mL expansion medium was added to each well and cultured in normoxic conditions. Culturing with the osteogenic medium began on day 3. Osteogenic induction medium was obtained by adding 100 nm dexamethasone, 10 mM β-glycerolphosphate, and 50 μg/mL ascorbic acid into the expansion medium [50]. The culture medium was renewed every 3 days during osteogenic induction.

*Seeding Efficiency and Cell Morphology*: To determine seeding efficiency, cultures were ended 24 h postseeding, and the samples were digested with activated papain enzyme digestion solution (APEDS):100 mM Sodium Phosphate Buffer/5 mM Na2EDTA/10 mM L-cysteine/3.88 units/mL papain (pH 6.5, all Sigma-Aldrich, St. Louis, MI, USA) at 60 °C under constant rotation for 18 h. To normalize seeding efficiency, the DNA content of the original seeding number was also measured (*n* = 4). DNA content was quantified using a Quant-iT™ PicoGreen™ dsDNA Assay Kit (Invitrogen, Waltham, MA, USA, P7589). Briefly, 10μL of each standard/sample solution was placed into individual wells in triplicate in a black, flat-bottomed 96-well plate, and 190 μL of Quant-iT™ PicoGreen™ dsDNA Assay solution (Molecular Probes, Biosciences) was added using a multichannel pipette. The plate was covered with aluminum foil and allowed to incubate for 5 mins at RT; it was read using a microplate reader (BioTek Instruments, Inc., Synergy HT Microplate Reader, Swindon, UK), with excitation and emission wavelengths of 485 nm and 528 nm, respectively.

*Cell Viability Assessments and Proliferation*: AlamarBlue™ (Invitrogen, Waltham, MA, USA) cell viability assay was performed to measure the metabolic activity of the cells seeded (0.5 × 10^6^ pBMSC/scaffold) on the PGS-deB blend scaffolds. The assay reagent was prepared according to the manufacturer’s procedure. Scaffolds were transferred to a 48-well plate (one scaffold/well), and a 1 mL assay dye solution was added to each scaffold incubated for 4 h at 37 °C in normoxic conditions by protecting them from light exposure. After incubation, 100 µL/sample of AlamarBlue media was pipetted in triplicates into a 96-well plate, and the fluorescence was measured immediately afterward on a plate reader (BioTek Instruments, Inc., Synergy HT) using excitation and emission wavelengths of 520 nm and 590 nm, respectively.

Proliferation was studied on days 3 and 21 and was determined using the DNA content quantified from a Quant-iT™ PicoGreen™ dsDNA Assay Kit (Invitrogen, Waltham, MA, USA, P7589), as aforementioned in the previous method section.

### 2.4. Characterization of Osteogenic Differentiation

*Extracellular ALP*: Extracellular ALP was quantified on days 3, 6, 15, and 18 of the culture period. A 1 mM p-Nitrophenyl phosphate (pNPP, Sigma N1891) solution was used to generate the serial dilutions to create a proper standard curve. The dilutions were applied with ddH_2_O, with either expansion (EM) or osteogenic (OM) medium used, depending on the medium the cells were in at a given time point. Each pNPP standard (120 µL) was added to a flat-bottomed 96-well plate, and 10 µL of 43 µM ALP Enzyme (Sigma P6774) solution was added just before 60 min incubation at 25 °C by light protection. For the test samples, 50 μL of 5 mM pNPP was added to the appropriate wells, with 40 μL of sample medium being added, followed by 40 μL of ddH_2_O. All reactions were stopped by adding 20 µL of 3 M NaOH (Sigma S8045) into each standard and sample, and subsequently, the plate was read at 405 nm. ALP activity was calculated as the amount of µM pNPP generated by the samples divided by the volume of sample and reaction time.

*Mineral Production*: Deposited calcium within the scaffolds was quantified with a calcium assay kit (Sentinel Diagnostics, Milano, Italy). Prior to analysis, the samples were digested overnight in 1 M HCL (1000 μL/sample) on a rotator at 12 rpm and 60 °C until no white precipitate remained. The working dye solution was prepared according to the manufacturer’s procedure. For the assay, 10 µL of each standard and sample were added into individual wells in triplicate in clear round-bottomed 96-well plates, and 140 µL of working dye solution was subsequently added, and the absorbance was read at 570 nm (Biotek, Synergy HT, Swindon, UK). An appropriate standard curve was generated, and the calcium contents were quantified. Each group’s calcium content on day 3 was subtracted from day 21 to eliminate the higher levels of calcium incorporated into the scaffolds via the decellularized bone.

*Histological Analyses:* Both constructs were cultured in an osteogenic differentiation medium for 21 days, and the 24 h cultured groups were cryogenically embedded as previously described [51]. Before cryoembedding, the samples were fixed with 4% paraformaldehyde (PFA) for 24 h in a 4 °C fridge and washed in PBS 3 times for 10 min each. Frozen samples were sectioned with a thickness of 30 µm using a cryostat (Leica), and the sections were preserved at 20 °C until staining. Before every staining, each section (3 section/glass slide) was covered with a liquid-blocker, and a 200 µL staining solution was used. After 5 min PBS washing, the sections were stained with a 1% alizarin red solution for Ca production (mineralization), and picrosirius red for collagen distribution, and finally visualized using the Aperio ScanScope slide scanner (assessed by using Aperio ImageScope and ImageJ software).

*In vitro degradation by size:* In vitro degradation by size was determined through the histological staining of the whole scaffolds. For this purpose, alizarin red stains of the scaffolds at day 1 and day 21 were used to measure the bidirectional diameter of the scaffolds (see Supplementary Materials).

### 2.5. Statistical Analyses

Statistical analyses were performed using GraphPad Prism 7.0 (San Diego, CA, USA), and the results were presented as mean ± standard deviation. All experiments were analyzed using one-way or two-way ANOVA where needed, followed by Tukey’s multiple comparison post-test. Statistically significant differences are denoted as * *p* < 0.05; ** *p* ≤ 0.01; *** *p* ≤ 0.001; **** *p* < 0.0001 and &, #, α symbols were used where needed.

## 3. Results

### 3.1. Characterization of the PGS-deB Blend Scaffolds

#### 3.1.1. Physicochemical Properties

**Figure 2:**
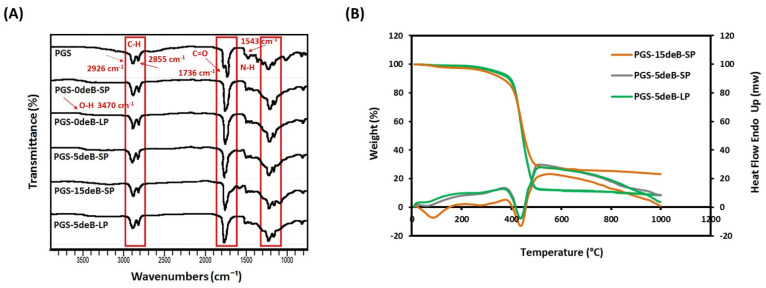
ATR-FTIR spectra: the cured PGS film, the porous plain scaffolds PGS-0deB-SP, and PGS-0deB-LP, and the decellularized bone incorporated scaffolds PGS-5deB-SP, PGS-15deB-LP, and PGS-5deB-LP (**A**); DSC/TGA thermograms of the PGS-deB blend scaffolds (**B**).

The ATR-FTIR spectrum of all the PGS-deB blend scaffolds (including the control PGS film) presented a broad absorption peak for the hydroxyl groups at 3470 cm^−1^, while the PGS-15deB-SP scaffold shifted to 3287 cm^−1^, indicating that the increased crosslinking density reduced the hydroxyl groups in the polymer backbone (Figure 2A) [30]. The stretch vibration of the methyl and alkane groups was observed as sharp peaks at 2926 cm^−1^ and 2855 cm^−1^, respectively [52]. The peaks at around 960 cm^−1^ and 1032 cm^−1^ could be assigned to the orthophosphate (PO_4_^3−^) group, reflecting the presence of nanocrystalline mineral as a result of bone ECM insertion into the scaffold, while the common peak observed at 1543 cm^−1^ in the bone ECM containing the scaffolds is a sign of protein amide II corresponding to the doped collagen into the scaffold [53]. The distinct peaks at 1736 cm^−1^ were detected in all of the PGS-deB blend scaffold groups, as well as in the PGS film, and are directly associated with the formation of ester bonds. Overall, all of the groups show symmetric spectra, indicating the insertion of decellularized bone into the PGS elastomer, with the signature maintained bands, like ester linkages, confirming the synthesized polymer is a polyester [54].

The TGA thermograms of the three different PGS-deB blend scaffolds exhibited single-step weight loss (Figure 2B). Significant weight loss occurred between around 380–470 °C for all of the blended scaffolds and also for the porous PGS scaffolds without bone ECM (PGS-0deB-SP and PGS-0deB-LP). Table 1 shows the decomposition temperatures of the PGS-deB scaffolds for different % weight loss (Tx; T10%, T25%, Tf) and the residual concentrations at the final decomposition temperature (Tf). Thermal stability was enhanced by the incorporation of decellularized bone ECM, notably in the small porous PGS-deB scaffolds (according to Tf values; PGS-0deB-SP: 490.500, PGS-5deB-SP: 492.749, and PGS-15deB-SP: 505.517, respectively [55]). A similar correlation could not be seen for the large porous scaffolds, yet the initial decomposition temperature (T10%) is higher in PGS-5deB-LP: 389.041 than in PGS-0deB-LP: 379.570. Although the initial decomposition temperature (T10%) is relatively low for the PGS-15deB-SP scaffold, it shows higher thermal stability than the other scaffolds. The inorganic components, originating from incorporated decellularized bone, remained constant above Tf. The residual inorganic concentration (wt%) in the scaffolds indicated that the decellularized bone blended well with the PGS.

The thermal behavior of the PGS-deB blend scaffolds was investigated by DSC (Figure 2B). The PGS-deB blend glass transition temperatures (Tg) were measured: 2.87 °C, 2.68 °C, and −3.90 °C for PGS-5deB-SP, PGS-5deB-LP, and PGS-15deB-SP, respectively. The melting transition temperatures ranged from −7.67 to 13.56, −7.54 to 12.03, and −12.88 to 5.27 °C for PGS-5deB-SP, PGS-5deB-LP, and PGS-15deB-SP, respectively. The DSC results show that the synthesized polymeric PGS-deB blends are amorphous at 37 °C [56].

#### 3.1.2. Scaffold Microarchitecture and Density

**Figure 3:**
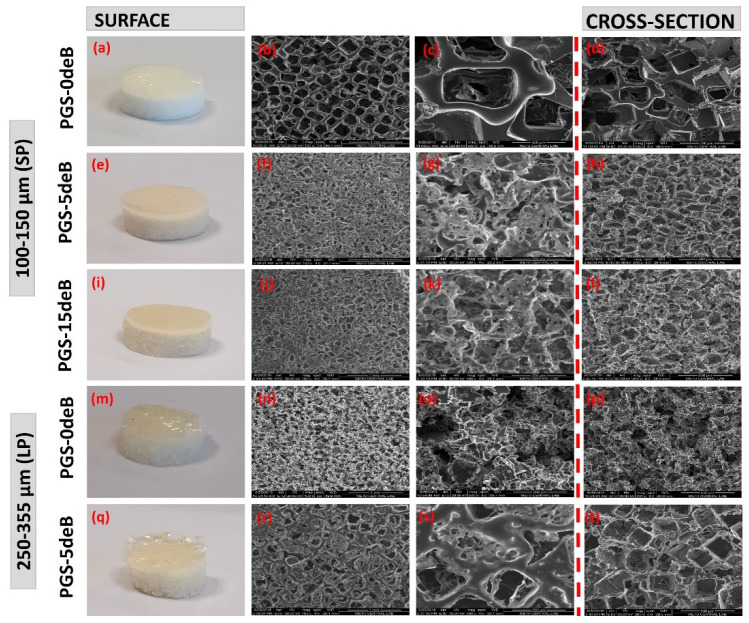
SEM micrographs of the PGS/deB blend scaffolds from the surface and cross-sectional areas. Please see the detail explanation of the subfigures (**a**–**t**) in the above paragraph.

**Figure 4:**
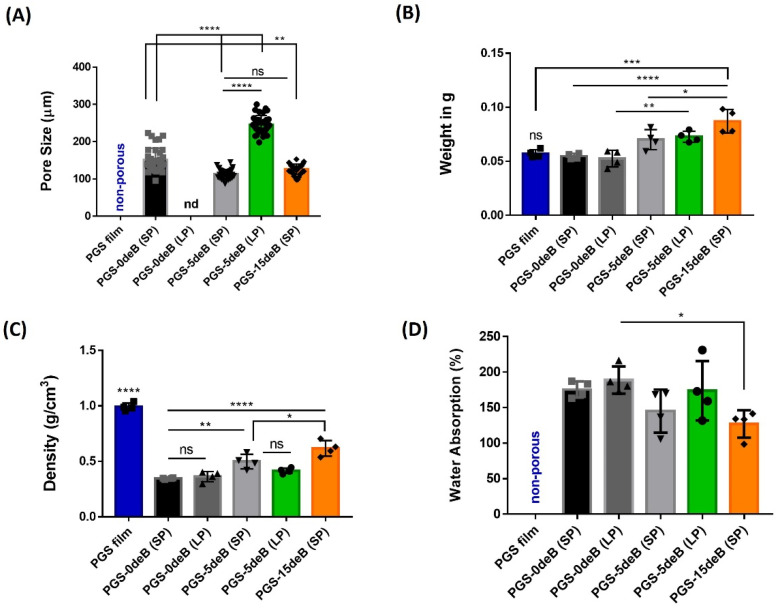
The pore size (μm) (**A**), weight (g) (**B**), density (g/cm^3^) (**C**), and water absorption (**D**) of all PGS-deB blend scaffolds, with small (SP) to large pores (LP), or with varying amounts of bone insertion. * *p* < 0.05; ** *p* ≤ 0.01; *** *p* ≤ 0.001; **** *p* < 0.0001 indicates significance. Each data have plotted as geometrical symbols on bars.

The microstructural evaluations produced by the SEM micrographs are presented in Figure 3. The final plain porous PGS scaffolds are characterized by an opaque white color, as seen in Figure 3a,m, whereas the decellularized bone inserted into the PGS-deB blend scaffolds showed a light brownish-yellow color, Figure 3e,i,q. All the scaffolds demonstrated highly interconnected and open-pore microstructures, yet there were obvious alterations between the scaffolds with and without the added bone material. The PGS-0deB-SP (0 wt% deB) scaffolds were characterized by a very distinct rectangular shape pore microarchitecture (Figure 3b–d) and featured an average pore size of 151.45 ± 31.32 μm (Table 2). In comparison, the PGS-5deB-SP (14 wt% deB) scaffolds presented rather thin pore struts (Figure 3f–h) with an average pore size of 107.73 ± 15.93 μm. Bone incorporation into the scaffold led to a thinning of the struts and also created a significant reduction in the average pore size (Figure 4A). Further increasing the amount of bone inserted into the scaffold (PGS-15deB-SP (28 wt% deB)) (Figure 3j–l) did not lead to a significant reduction in the average pore size. Scaffold pore size was analyzed using Image J software, which revealed that PGS-5deB-LP had the largest pore size (Figure 3r–t) of 246.79 ± 26.58 μm. The bone-free large porous scaffolds (PGS-0deB-LP) could not be accurately measured due to having an undefined pore structure (Figure 3n–p) (Table 2: nd: not detectable). A very thin layer of PGS film of between 100–400 μm in thickness formed on the top of the scaffold following DHT crosslinking, likely due to low vacuum (8 mbar) exposure. In Figure 3, all SEM images were obtained from the surface of the bottom layer or cross-section of the entire scaffold to determine the porous structure. There were no significant differences in porosity between any of the other scaffolds, either bone-inserted or not, and the porosity of the scaffolds mostly fitted into the designed range, except the PGS-15deB-SP and PGS-5deB-LP scaffolds, which measured 71.30 ± 6.27 % and 71.39 ± 9.22 %, respectively (Table 2).

The measured pore size of the scaffolds, the volume ratio of PGS/NaCl/deB (VPGS:VNaCl:VdeB), and the weight ratio of PGS/deB in the final blend scaffold (wt% PGS: wt% deB), as well as the porosities, are also given at Table 2. Since the used polymer mass was constant for all the scaffolds, the weight of the PGS film, PGS-0deB-SP, and PGS-0deB-LP are almost similar, as seen in Figure 4B. As a consequence of bone insertion, the weight of the PGS-15deB-SP blend scaffold significantly increased. It was determined that the density of all of the porous tissue scaffolds was statistically lower than the PGS film at 0.9904 g/cm^3^ (Figure 4C). However, the PGS-deB blends became denser due to bone incorporation. The PGS-15deB-SP scaffolds with the highest bone appeared denser (0.6165 g/cm^3^) than the other blends but were also found to be the scaffold type with the closest density to the PGS film. Figure 4D shows the water absorption (%) of the scaffolds following 17 h of immersion. It was observed that absorption decreased proportionally with increasing amounts of incorporated bone. The PGS-0deB-SP scaffold absorbed 175% water, while PGS-5deB-SP absorbed 145.1%, and PGS-15deB-SP absorbed 126.8%. The same situation was observed in the large-porous tissue scaffolds. The bone-free scaffold with large pores (PGS-0deB-LP) induced the highest water uptake ratio (189%), while water absorption diminished (to 173.6%) with the addition of bone into the structure (PGS-5deB-LP). There was no statistical difference in terms of the water absorption ratio between the small- and large-porous bone-free scaffolds.

#### 3.1.3. Degradation/pH Change of the PGS/deB Blend Scaffolds

**Figure 5:**
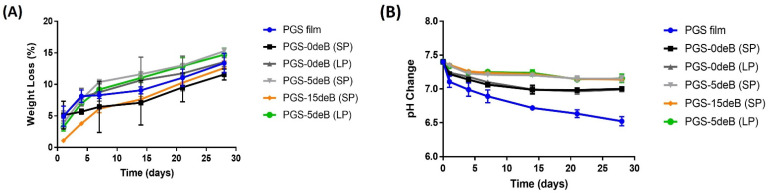
The degradation (**A**) and pH change (**B**) of physiological Tris–HCl buffer regarding the PGS-deB blend scaffolds and PGS film.

The degradation profile of all scaffolds over 28 days was measured in a Tris-HCl buffer solution (pH = 7.4) (Figure 5A). Although there was no statistical difference between the scaffolds in terms of degradation on the first day, the PGS-15deB-SP scaffold stands out with the lowest degradation rate: 1.076 ± 0.22%. Mass loss appeared to decrease with the further addition of bone into the small-pore scaffold (PGS-0deB-SP). While the mass loss was 5.127% in the PGS-0deB-SP scaffold on the first day, it decreased to 3.862% in the PGS-5deB-SP scaffold and 1.076% in the PGS-15deB-SP. The same trend was observed in the large-porous scaffolds. The degradation rate of the PGS-deB blends, as well as the PGS film, exhibited a constant and linear increase over the 28 days of the incubation period. At the end of this period, the PGS-0deB-SP and the PGS-15deB-SP scaffolds demonstrated the lowest degradation rates: 11.58% and 12.59% in 28 days, respectively.

During the degradation period, the pH values were recorded for the same, defined time points. Figure 5B shows the change in pH of the physiological Tris–HCl buffer over 28 days. It was determined that the pH value of the PGS film sharply reduced from 7.4 to 7.10 ± 0.084 (*n* = 4) on the first day of incubation. However, for the porous tissue scaffolds, whether bone-free or bone-inserted, a significant difference was observed for the pH change on the first day, compared to the PGS film. More importantly, the pH of all the bone-inserted PGS-deB blend scaffolds was maintained almost constantly during the first day, measuring around 7.35 on average for all three of PGS-deB scaffolds. The pH value of bone-inserted scaffolds decreased more slowly than the PGS film over 28 days.

#### 3.1.4. Bone Incorporation Enhances the Crosslinking Density and Hydrophilicity of the PGS-deB Blend Scaffolds

**Figure 6:**
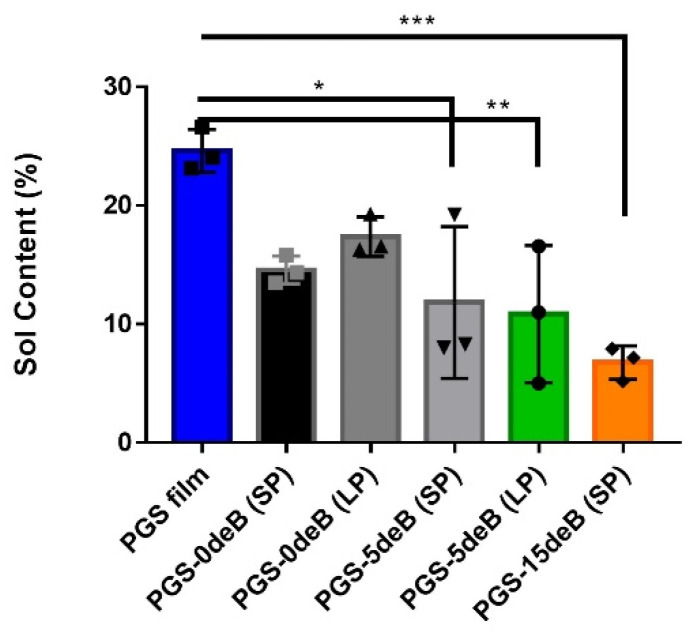
The crosslinking density of all the PGS-deB blend scaffolds and PGS film; the sol fractions % (non-crosslinked content) of the PGS film and PGS-deB blend scaffolds were determined via sol–gel content; * *p* < 0.05; ** *p* ≤ 0.01 and *** *p* ≤ 0.001 indicates statistical significance and each data have plotted as geometrical symbols on bars (n = 3).

In order to determine the crosslinking density of the scaffolds, the sol fractions (uncrosslinked macromer) and gel content (crosslinked network) were measured (Figure 6 and Table 3). Both scaffold porosity and the insertion of bone influenced the sol fractions and the extent of the crosslinking.

**Figure 7:**
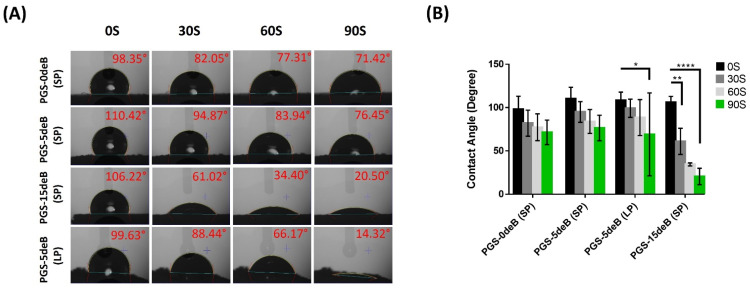
Hydrophilicity measurements of the PGS-deB and bone-free porous PGS scaffolds were taken at 0, 30, 60, and 90 s of dropping ddH_2_O onto the surface (**A**); the measured values are shown as mean ± standard deviation and * *p* < 0.05; ** *p* ≤ 0.01 and **** *p* < 0.0001 indicates statistical significance (*n* = 4) (**B**).

The hydrophilicity of the PGS-deB and bone-free porous PGS scaffolds was also assessed at 0, 30, 60, and 90 s by dropping ddH_2_O onto their surfaces (Figure 7). In the PGS-15deB-SP scaffold group, the accelerated uptake of water was observed notably in the first 30 s in comparison with the other groups, and the water contact angle sharply reduced to 61.02 ± 15.06° from 106.22 ± 6.77°. It was determined that the PGS-deB scaffolds exhibited a hydrophilic character proportional to the increasing amount of decellularized bone in the material structure. The water contact angle of the nonporous PGS film was measured as 66.72 ± 5.11° at 60 s (*n* = 4, data not shown), which was higher than that of PGS-15deB-SP (34.40 ± 1.80°), demonstrating that bone inclusion enhanced hydrophilicity.

#### 3.1.5. Bone Incorporation Enhances the Mechanical Stiffness of the PGS-deB Blend Scaffolds

**Figure 8:**
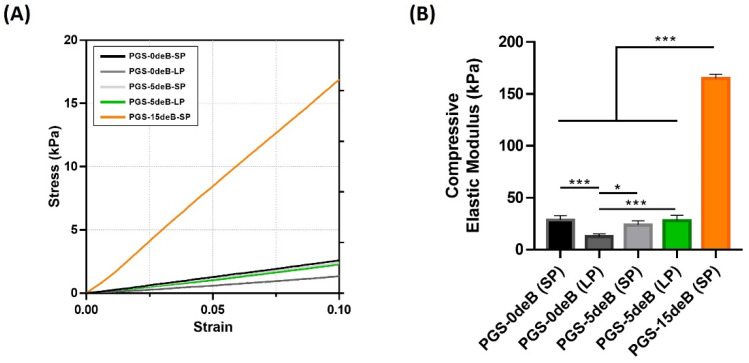
The effects of deB incorporation and pore size on the mechanical properties of the PGS-deB blend scaffolds and bone-free porous PGS-0deB-SP and PGS-0deB-LP; stress-strain curve (**A**) and compressive modulus; * *p* < 0.05 and *** *p* ≤ 0.001 indicates statistical significance (**B**).

The compressive Young modulus (Ec) of all the groups was obtained from the slope of the obtained stress-strain curves in kPa (Figure 8B). The compressive young modulus of the small porous bone-free scaffold (PGS-0deB-SP) was significantly higher than that of the large porous bone-free scaffold (PGS-0deB (LP)). It was also determined that the presence of the decellularized bone in the PGS-deB blend scaffolds increased the elastic modulus of the structure by up to 164.9 kPa in the PGS-15deB-SP scaffolds. The Young modulus of the small pore scaffolds (28.59 kPa) increased 5.7-fold with the insertion of 28 wt% decellularized bone into the construct. However, there were no differences in the Young modulus of the PGS-0deB-SP and PGS-5deB-SP scaffolds. It should be noted that, whether bone-inserted or bone-free, all the scaffolds withstood high compression and exhibited full shape recovery following the release of load.

### 3.2. Osteogenic Differentiation Potential of Isolated pBMSCs

**Figure 9:**
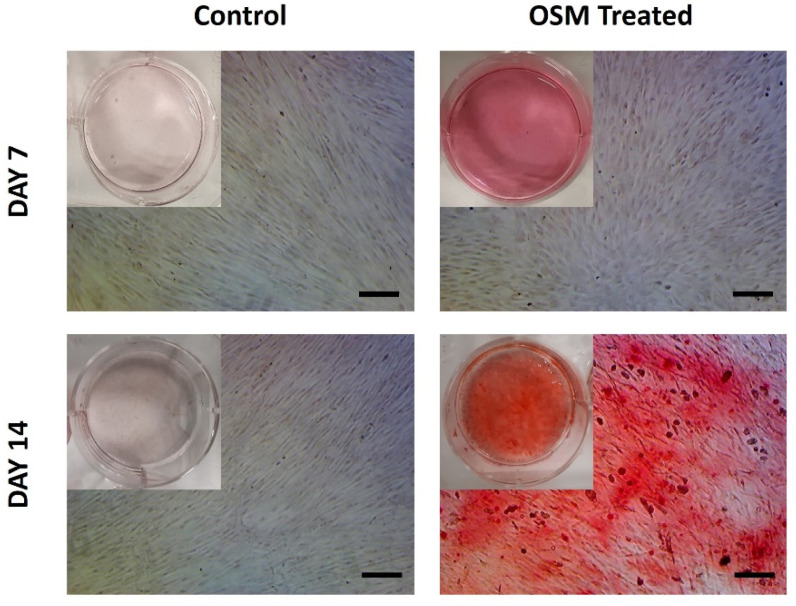
Determination of the osteogenic differentiation potential of the isolated pBMSCs; alizarin red staining of monolayer pBMSCs treated with XPAN and OSM on days 7 and 14 of the culture (Scale: 250 μm). Macroscopic images of the ARS-stained wells are also presented within the cell images.

The osteogenic differentiation potential of the isolated pBMSCs was determined using the 2D surface of a 6-well plate. Both the control (negative) and the OSM-treated (positive) monolayer cells were stained with an ARS solution on days 7 and 14 of the culture. The deposited calcium formed an alizarin red S-calcium complex at day 14 in the OSM-treated cells, appearing as an orange-red color (Figure 9). In contrast, the control cells that were cultured with XPAN medium showed negative alizarin red staining at days 7 and 14, which can also be seen in the macroscopic images, showing a clear background. As a result of alizarin red staining, we demonstrated that the isolated pBMSCs had osteogenic differentiation potential on a 2D surface.

### 3.3. Attachment, Activity, and Proliferation of pBMSCs

**Figure 10:**
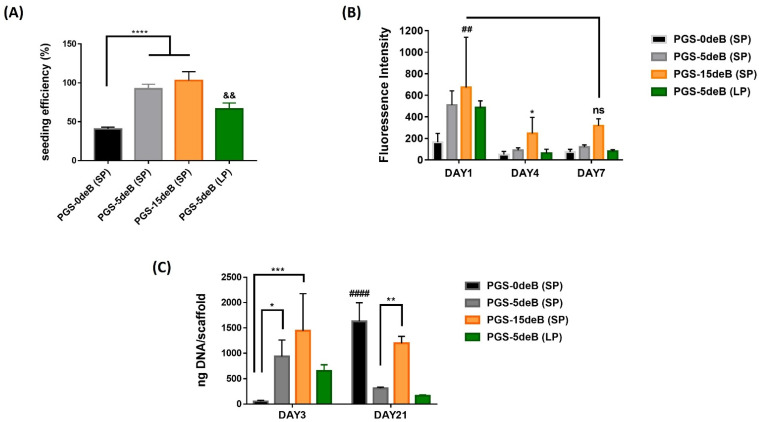
Seeding efficiency (%) quantified via DNA content measurement; **** *p* < 0.0001 statistical significance and ^&&^ *p* ≤ 0.01 for PGS-5deB-SP vs PGS-5deB-LP (**A**); cell viability, determined with AlamarBlue cell viability assay; ^##^ *p* ≤ 0.01 for DAY1: PGS-0deB (SP) vs. DAY1: PGS-15deB (SP) and * *p* < 0.05; ** p ≤ 0.01; *** p ≤ 0.001 for DAY1: PGS-15deB (SP) vs. DAY4: PGS-15deB (SP) (**B**), and proliferation were determined on days 3 and 21 of the culture; ^####^ *p* < 0.0001 for DAY 3: PGS-0deB (SP) vs. DAY 21: PGS-0deB (SP) (**C**).

Bone incorporation into the scaffold can clearly be seen to influence cellular attachment onto the PGS scaffolds (Figure 10A). Seeding efficiency was significantly enhanced by the insertion of decellularized bone tissue, and the greatest seeding efficiency was achieved in the small-porous highest bone-doped scaffold (PGS-15deB-SP). Besides that, there was a significant difference between the small- and large-porous scaffolds in terms of cell seeding efficiency. While seeding efficiency was determined as 92.35% for the PGS-5deB-SP scaffold, it was reduced to 66.26% for the PGS-5deB-LP scaffold. We did not perform this for the bone-free large pore scaffold as a control group since PGS-5deB-LP has a low cell attachment ratio despite the bone incorporation into the scaffold. It is also worth noting that, for the biological assessment of the scaffolds, we did not remove the top thin PGS film layer to avoid the manipulation of the construct; instead, we turned the upside of the scaffold down and seeded the cells on this surface.

The same trend in attachment was also seen in the cellular metabolic activity 24 h postseeding (Figure 10B). The highest cell activity was seen in the PGS-15deB (SP) scaffolds on day 1, which was significantly higher compared to the bone-free small porous scaffolds (PGS-0deB (SP) vs. PGS-15deB (SP)). The metabolic activity of the cells was significantly reduced on day 4 in all groups. Yet cellular activity started to increase in the PGS-15deB-SP scaffolds on day 7.

Proliferation was studied for 21 days, and the DNA content was determined on days 3 and 21 (Figure 10C). Even though the attachment rates were high, notably for the bone-inserted small-pore scaffolds, a significant increase in DNA content with culture time was not observed for the PGS-deB blend scaffolds.

### 3.4. Morphological Evaluations of the Cells Seeded on the PGS-deB Blend Scaffolds

**Figure 11:**
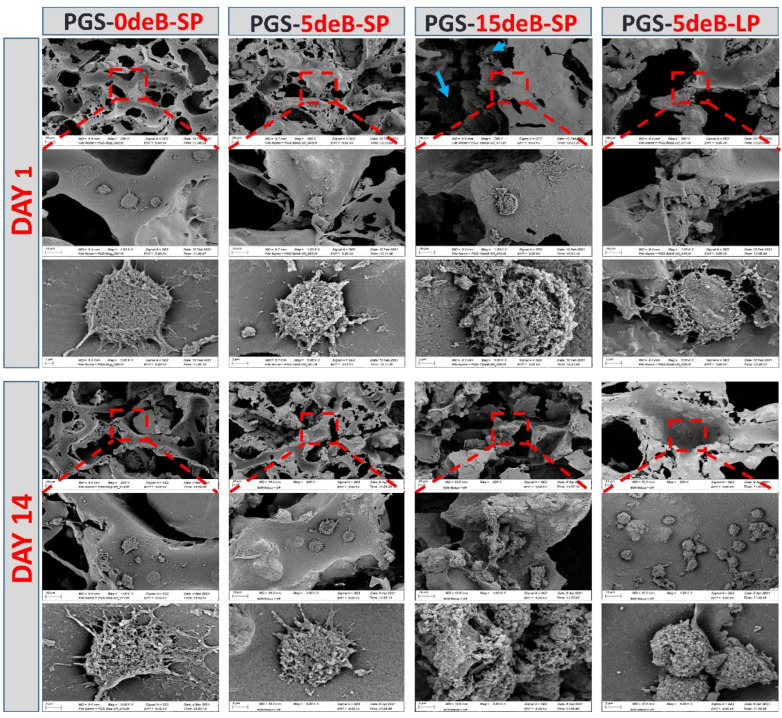
SEM micrographs of the cell-seeded constructs on days 1 and 14 at different magnifications. Red dotted boxes indicate the regions of interest (ROIs) and blue arrows show cells located in a certain depth of PGS-15de-SP scaffolds at DAY 1.

Cell attachment, observed via SEM imaging, revealed that the pBMSCs adhered and showed a spread-out and flat cell morphology on all scaffolds on day 1 postseeding (Figure 11). The cell–scaffold connections were better observed on day 1, and the cells integrated well with the surrounding scaffold in all of the compositions. Good integration between the cells and scaffolds was maintained up until day 14 in all blends except PGS-5deB-LP, for which the cells partly adopted a more rounded state.

### 3.5. Osteogenesis of pBMSCs in the PGS-deB Blends

**Figure 12:**
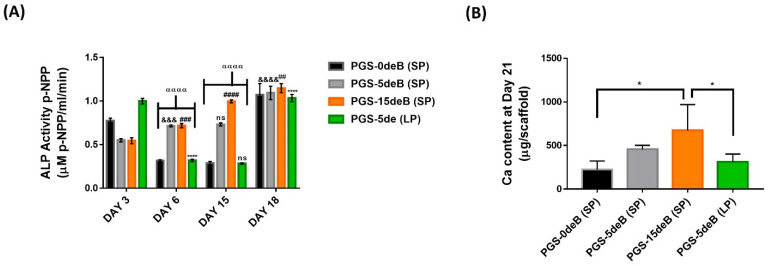
ALP activity (μM p-NPP) in the PGS-deB blend scaffolds on days 3, 6, 15, and 18 of the culture under osteogenic induction; *n* = 4, ^&&&^, ^&&&&^, ^##^, ^###^, ^αααα^, ^*^ and ^****^ = statistical significance via two-way ANOVA multiple comparisons between each scaffold and it’s former day point (^&&&^ *p* ≤ 0.001 for Day 3 PGS-5deB-SP vs. Day 6 PGS-5deB-SP or ^&&&&^ *p* < 0.0001 for Day 15 PGS-5deB-SP vs. Day 18 PGS-5deB-SP) and also between different groups (^αααα^ *p* < 0.0001) (**A**). Deposited Ca content within the scaffolds over a 21-day culture period (day 3 Ca content subtracted from day 21 to eliminate the higher levels of calcium incorporated into the scaffolds via decellularized bone); *n* = 4, * is statistical significance via one-way ANOVA multiple comparisons between each scaffold group (**B**).

ALP expression was determined quantitatively as an early bone formation marker [57,58]. Notably, in PGS-15deB-SP, ALP expression was found to increase continuously over the course of the culture, with a two-fold increase on day 15 compared to day 3, and a slight increase was also found from day 15 to 18 (Figure 12A). Likewise, there was an increase in the expression of ALP in the other bone-inserted scaffolds, PGS-5deB-SP, but ALP expression remained constant between days 6 and 15. There was a sudden and sharp decrease in ALP expression between days 3 and 6 in both the bone-free (PGS-0deB-SP) and large-pore scaffolds (PGS-5deB-LP). Lastly, no increase was observed on day 15 for both the PGS-0deB-SP and PGS-5deB-LP groups.

In order to determine the extent of scaffold mineralization, the Ca content was quantified as a late marker of osteogenic differentiation (Figure 12B) [12]. Calcium deposition increased in proportion to the amount of the incorporated decellularized bone, which is consistent with the previous cell-seeding efficiency results. There was significantly more calcium deposited in both PGS-5deB-SP (456.47 μg) and PGS-15deB-SP (672.41 μg) when compared to their mineral-free counterparts (PGS-0deB-SP: 224.21 μg Ca). There was no significant difference in calcium deposition between the small- and large-porous PGS-deB scaffolds containing the same amount of decellularized bone (PGS-5deB-SP: 456.47 µg vs. PGS-5deB-LP: 311.55 µg; ns).

**Figure 13:**
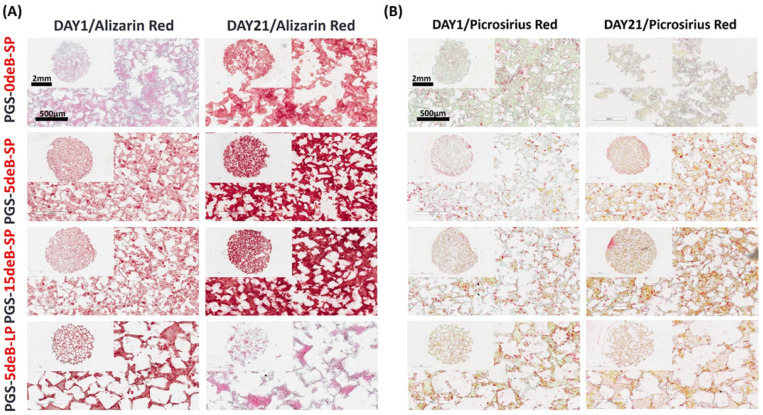
Mineralization was determined with alizarin red staining (**A**), and collagen production was evaluated with picro sirius red staining on days 1 and 21 of the culture (**B**).

Mineralization was further verified by alizarin red staining (Figure 13A). Notably, higher calcium deposits were detected in the PGS-deB blended scaffolds, particularly in the bone-inserted small-porous scaffolds, PGS-5deB-SP and PGS-15deB-SP. In comparison to their small-pore counterparts, fewer calcium deposits were detected in the large-pore scaffolds on day 21 of in vitro culture (PGS-5deB-LP). Picrosirius red staining shows that the collagen network became slightly denser in the PGS-5deB-SP and PGS-15deB-SP scaffolds with culture time. Similar levels of collagen production could not be observed in the PGS-5deB-LP scaffolds on day 21 (Figure 13B).

## 4. Discussion

The goal of this study was to develop PGS scaffolds, for the first time, that were functionalized with a decellularized bone matrix for employment in BTE. Specifically, we fabricated PGS/deB scaffolds with different amounts of incorporated bone ECM. Additionally, small- and large-pore size was compared in an attempt to optimize the internal architecture of the bone scaffold. Finally, we evaluated in vitro osteogenesis by seeding these scaffolds with bone marrow-derived mesenchymal stem cells (BMSCs). The results of this study demonstrated that the PGS polymeric structure could be tailored properly in terms of both pore size and bone ECM incorporation to develop scaffolds that were supportive of the osteogenic lineage commitment of MSCs. Furthermore, the mechanical strength and stiffness were significantly enhanced by bone ECM incorporation without compromising the elastomeric property of the final construct.

We began by demonstrating the successful incorporation of decellularized bone (deB) ECM into PGS elastomer while preserving its elastomeric nature, as evident by the signature peaks in the FTIR spectrum. Moreover, the synthesis route also preserved the bone ECM constituents, which did not denature in DHT crosslinking. Bone ECM incorporation was found to improve the thermal stabilization of the scaffolds, particularly in the small-pore scaffolds. Namely, it takes a longer time and a higher temperature to decompose the crosslinked small-pore highest bone-inserted scaffolds than their large-pore counterparts. [59,60]. Enhanced thermal behavior with increasing bone insertion could be a consequence of improved crosslinking density, bringing about higher decomposition temperatures. Kerativitayanan et al. also reported that the incorporation of nanosilicates enhanced the thermal stability of PGS-nanosilicate nanocomposite scaffolds [61].

Since the scaffold microarchitecture alone can drive the cells in a specific lineage, and features like porosity, pore size, and geometry have a direct influence on bone tissue regeneration [14,62], we examined the PGS-deB scaffold microarchitecture by SEM imaging. The bone-free small-pore scaffolds (PGS-0deB-SP) had very distinctive rectangular pore geometry and thick vertical struts, probably as a consequence of the solvent-free casting leading to a weak dispersion of pre-PGS into the mold. Frydrych et al. also reported that thick struts are an indicator of poor polymer dispersion in PGS/poly(L-lactic acid)(PLLA) blend scaffolds [63]. However, bone inclusion exhibited a direct influence on pore geometry and strut thickness by compensating for the consequences of solvent-free casting, as seen in the bone-free scaffolds. This is probably because reducing the number of salt crystals and replacing them with bone ECM during the scaffold fabrication process may have resulted in better dispersion of the prepolymer.

The PGS film exhibited a hydrophilic nature due to the presence of hydroxyl groups in the polymer backbone and, therefore, reduced the water contact angle. However, bone incorporation into the structure was found to improve scaffold hydrophilicity. Improved hydrophilicity via the incorporation of demineralized and decellularized bone ECM into the scaffolds has been also demonstrated [44]. In conclusion, the water contact angle results indicate that increased levels of bone powder can potentially support blood filling into implanted scaffolds and enhance cellular attachment.

Scaffold degradation is a crucial characteristic that needs to be considered, in particular, for polyester-based tissue regeneration applications, since degradation rate and byproducts have a direct effect on long-term regeneration and tissue formation [64,65]. The degradation mechanism of PGS is based on ester chain scission from the material surface by hydrolytic erosion [66], and both precursors, sebacic acid and glycerol, are natural constituents of the body [37,56]. The results in this study show that bone incorporation into PGS provided the opportunity to tailor degradation behavior. Both the PGS-deB blends and the PGS film exhibited superior degradation behavior compared to the literature [67]. Herein, the degree of crosslinking is likely a more prominent reason for having a better degradation profile. Since there is no significant difference between the 14% bone-inserted SP and LP scaffolds in terms of crosslinking density, a similar mass loss was observed. However, the 28% bone-incorporated small-pore constructs enhanced the crosslinking density by up to 93.25% (PGS-15deB-SP), leading to the lower degradation of the scaffold. Although the exact mechanism of action is not clear, a possible reason may be that the bone ECM in the structure interacted with the free hydroxyl groups in the PGS backbone, thus increasing the crosslinking density. Another possible reason might be due to the extension of surface area from the bone in the structure, thereby enhancing esterification. In vitro degradation via size measurement showed that no significant degradation had occurred in the bone-incorporated scaffolds during 21 days of culture, except in the bone-free counterpart (see Supplementary Materials).

The addition of decellularized bone ECM into PGS was found to enhance cellular attachment significantly, particularly in the small-pore constructs. As demonstrated by many studies, the addition of ECM resulted in higher cell attachment in comparison with the ECM-free counterparts [41,43,44,68]. This could be due to the presence of collagenous/noncollagenous proteins, tissue-specific growth factors (known to promote cellular attachment [69]), or due to calcium ion release [70,71] and tailored surface topography [72,73]. Freeman et al. reported that decellularized bone matrix (DCB) incorporation in between 3D-printed PCL filaments drastically increased the attachment of porcine BMSCs in vitro and resulted in favorable vascularization and new bone formation in vivo [43]. The results indicated that pore size also had a significant effect on cellular attachment, with a reduction seen in the large-pore scaffold compared to the small-pore counterpart. This is associated with increased surface area in a small-pore structure, resulting in better initial cell attachment [26]. Despite having high initial cell attachment rates, cell viability decreased, and accordingly, proliferation did not increase. However, cell viability started to recover in the highest bone-inserted (PGS-15deB-SP) scaffold, and the cell number was preserved over the entire culture period.

Cell spreading shape and area have been shown to be a regulator of the osteogenic differentiation of MSCs and also determine their survival. Zhao Y et al. demonstrated that cell spreading is advantageous for the osteogenic lineage commitment of MSCs [74]. Consistent with these findings, the results in our study showed that porcine BMSCs were well spread on the small-pore scaffolds, as seen in the SEM micrographs. In contrast to the small-pore scaffolds, the cells displayed a rather circular shape on the large-pore constructs on day 14, correlating with the relatively weak osteogenesis of MSCs.

Small-pore scaffolds better support the osteogenic differentiation of MSCs. This was evident from increased calcium deposition, increased ALP expression, and positive alizarin red staining. Since the calcium-rich decellularized matrix incorporated scaffold had also been stained with alizarin red, the scaffolds were stained at the very beginning of the culture and also at the end of the culture period to assess mineralization. The scaffolds, even on day 1, were weakly stained with alizarin red but were also quite densely stained with alizarin red on day 21. As an attempt to avoid the possibility of misevaluation, quantitative measurements of the produced calcium amount were also performed following 21 days of culture. For this purpose, the Ca-rich scaffolds were fully digested before the culture and were subtracted from the initial high Ca amount from day 21 to eliminate the higher levels of calcium incorporated into the scaffolds via decellularized bone.

It has been reported that Ca deposition and ALP expression are enhanced in porous scaffolds with a relatively small pore size [75]. The effect of pore size on MSC differentiation can be different depending on the polymer, as well as the scaffold type used, which should be considered in the experimental design. Table 4 shows superior osteogenesis in scaffolds with different pore sizes fabricated from various materials. As seen in Table 4, superior osteogenesis differs greatly depending on the manufactured scaffold. The role of pore size on the scaffolds and the mechanism of action on its osteoinductivity is still not clear [76].

Although higher levels of initial cell attachment affect the ensuing events, such as proliferation, migration, and differentiation, there is evidence that the osteogenesis of pBMSCs within bone-inserted small-pore scaffolds is not mediated solely by initial cell attachment, but possibly also by regulatory factors present in bone ECM-containing scaffolds.

## 5. Conclusions

In summary, we have functionalized PGS with decellularized bone ECM and demonstrated the ability of the blend to support the osteogenesis of MSCs. Furthermore, we also investigated and demonstrated an intricate interplay between pore size and PGS elastomer composition in the context of osteogenic differentiation. The decellularized bone ECM functionalization of PGS not only mechanically enhanced the scaffold but also significantly increased the hydrophilicity of the structure. Accordingly, PGS scaffolds functionalized with a small pore size (100–150 μm) and relatively high levels (28%) of bone ECM incorporation best support the osteogenesis of MSCs and significantly increase mineralization. Besides, they enabled the highest seeding efficiency and cell spreading shape, which are favorable for osteogenic differentiation. Overall, a tailored PGS-based scaffold with small pore size and bone ECM could potentially be used for BTE applications.

## Figures and Tables

**Table 1:** Concentration of decellularized bone (deB) in the PGS/deB blend scaffolds, and the decomposition temperatures (Tx and Tf).

Sample	Design Specification (wt%)	Decomposition Temperatures (T10%; T25%; Tf) (°C)	Residue Concentration at Tf (wt%)
PGS-0deB-SP	0	362.75; 408.25; 490.50	2.913
PGS-5deB-SP	14	396.033; 422.675; 492.749	13.466
PGS-15deB-SP	28	362.745; 420.491; 505.517	28.822
PGS-0deB-LP	0	379.57; 422.21; 500.15	3.056
PGS-5deB-LP	14	389.041; 422.189; 495.293	13.849

**Table 2:** Pore size, volume, weight ratios, and porosities of the PGS/deB scaffolds.

Sample Code	Pore Size (μm)	PGS/NaCl/deB Volume Ratio *V*_PGS_:*V*_NaCl_:*V*_deB_	PGS/deB Weight Ratio wt%_PGS_:wt%_deB_ (in Final Scaffold)	Porosity (%)
PGS-0deB-SP	151.45 ± 31.32	30:70:0	-	65.67 ± 1.38
PGS-0deB-LP	nd	30:70:0	-	64.67 ± 4.45
PGS-5deB-SP	107.73 ± 15.93	30:65:5	87:13	65.37 ± 0.81
PGS-5deB-LP	246.79 ± 26.58	30:65:5	87:13	71.39 ± 9.22
PGS-15deB-SP	138.32 ± 19.42	30:55:15	72:28	71.30 ± 6.27

**Table 3:** Sol–Gel Content.

Sample	Sol (wt%)	Gel (wt%)
PGS	24.59	75.40
PGS-0deB-SP	14.56	85.43
PGS-0deB-LP	17.37	82.62
PGS-5deB-SP	11.82	88.18
PGS-5deB-LP	10.85	89.15
PGS-15deB-SP	6.755	93.25

**Table 4:** Superior osteogenesis in different pore size scaffolds.

Material Type	Cell Type	Superior Osteogenesis Pore Size
PEOT/PBT and PCL	hMSCs	Gradient (500 μm to 1000 μm) [77]
PCL	hMSCs	Small (100 μm) [78]
PCL	hOB	Gradient + Heterogenous offset (250 μm–500 μm–750 μm) [75]
Ti-6Al-4V	MC3T3-E1	Small (300 μm) [79]
HAp	BMSCs	Large (1300 μm-800 μm) [80]
PCL + DCB	BMSCs	Small (800 μm) and Large (1200 μm) * [43]

* ns: not significant difference between.

## Data Availability

Not applicable.

## Supplementary Materials

The following Supporting information can be downloaded at: https://www.mdpi.com/article/10.3390/bioengineering10010030/s1, Figure S1: In-vitro degradation by size measurements via alizarin red staining of whole scaffold at day 1 and day 21 (n = 3). Aperio ImageScope were used to determine the bilateral diameter of the whole scaffold. Figure S2: In-vitro degradation by size following 21 days culture period obtained via histological staining of whole scaffold (SF1 above) at day 1 and day 21 (n = 3), * = statistical significance via two-way ANOVA multiple comparisons between each scaffold with its former day point.
