# Supplementary material for: Biofabrication of Poly(glycerol sebacate) Scaffolds Functionalized with a Decellularized Bone Extracellular Matrix for Bone Tissue Engineering
Source: Bioengineering (Basel). 2022 Dec 25;10(1):30. doi: 10.3390/bioengineering10010030 (PMC9854839; doi:10.3390/bioengineering10010030)
Supplement: Supplementary file 1 [file bioengineering-10-00030-s001.zip › bioengineering-1992606-supplementary.pdf]

Supporting Information Supplement Figure S1:

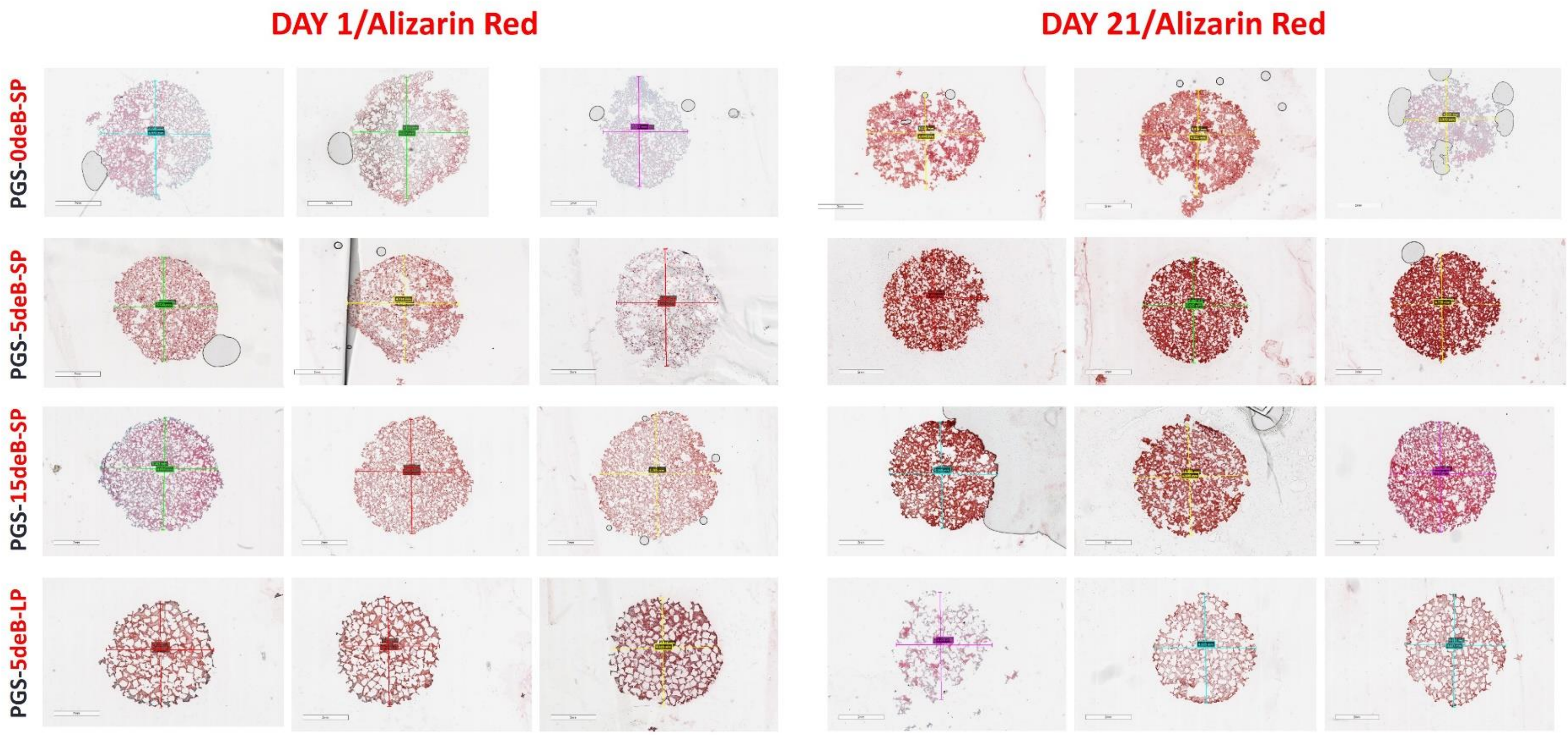

SF1. In-vitro degradation by size measurements via alizarin red staining of whole scaffold at day 1 and day 21 (n=3). Aperio ImageScope were used to determine the bilateral diameter of the whole scaffold.

Supplement Figure S2:

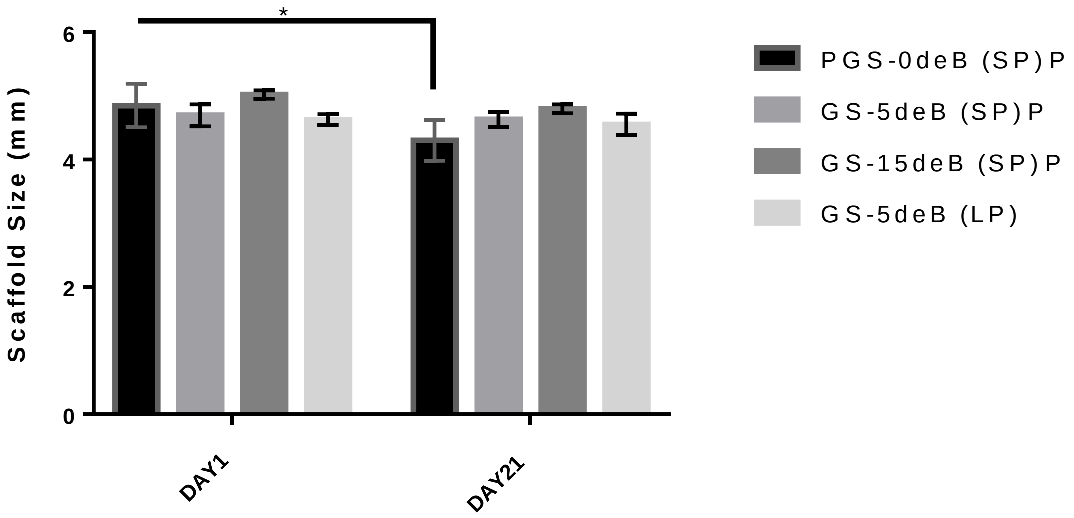

SF2. In-vitro degradation by size following 21 days culture period obtained via histological staining of whole scaffold (SF1 above) at day 1 and day 21 (n=3), * = statistical significance via two-way ANOVA multiple comparisons between each scaffold with its former day point.
